# Development of consensus best treatment plans for new-onset systemic juvenile idiopathic arthritis

**DOI:** 10.1186/1546-0096-10-S1-A50

**Published:** 2012-07-13

**Authors:** Esi M Morgan DeWitt, Timothy Beukelman, Peter A Nigrovic, Karen Onel, Sampath Prahalad, Rayfel Schneider, Matthew Stoll, Carol A Wallace, Yukiko Kimura

**Affiliations:** 1 Children's Hospital of Boston, Boston, MA, USA; 2 Childrens Hospital Regional Medical Center, Seattle, WA, USA; 3 Cincinnati Children's Hospital, Cincinnati, OH, USA; 4 Emory Children's Center, Atlanta, GA, USA; 5 Hackensack University Medical Center, Hackensack, NJ, USA; 6 Hospital for Sick Children, Toronto, ON, Canada; 7 University of Alabama-Birmingham, Birmingham, AL, USA; 8 University of Chicago, Chicago, IL, USA; 9 UT Southwestern Medical Center, Dallas, TX, USA

## Purpose

Currently, there is significant variability in the therapeutic approaches to new onset systemic Juvenile Idiopathic Arthritis (sJIA), as evidenced by surveys using case presentations administered to Childhood Arthritis and Rheumatology Research Alliance (CARRA) members. Understanding the comparative effectiveness of these diverse therapeutic approaches would likely result in better health outcomes. We therefore aimed to derive consensus based treatment plans, standardized assessment intervals and data collection plans for clinical use to facilitate comparative effectiveness studies for new-onset sJIA. Eligible patients may be enrolled into any of the treatment plans at the treating physician’s discretion.

## Methods

To develop eligibility criteria and consensus approaches to therapeutic management, the CARRA sJIA Core Workgroup convened regularly for workgroup meetings, hosted a 2-day CARRA consensus conference in April 2010, and conducted surveys of the CARRA membership.

## Results

The CARRA sJIA Core Workgroup developed formal eligibility criteria for patient enrollment, standardized treatment approaches, and a recommended schedule of visits and monitoring parameters. Entry criteria include age 6m-18y and probable sJIA determined by defined criteria. The treatment regimens include: 1) a corticosteroid based plan, 2) a methotrexate based plan – with/without corticosteroid, and 3) an anti-IL1 (anakinra) based plan – with/without corticosteroid. A survey of the CARRA membership was conducted in December 2010 to assess the acceptability and feasibility of these strategies. There was a 63% response rate (133 of 211 surveyed), of which 92.6% expressed willingness to follow 1 of 3 treatment plans as outlined. 82% concurred that a 4th anti-IL6 based treatment arm should also be offered. Consensus was reached at the 78-85% level for all topics posed (entry criteria, specific details of treatment plans, ability to use plans). A feasibility study estimated that over 250 patients could be enrolled in these plans per year, and that physicians would likely enroll one-third to one-quarter of the patients in each of the original 3 plans.

## Conclusion

The use of consensus derived standardized treatment plans for new onset SJIA is feasible and acceptable to most North American rheumatologists who are members of CARRA. Four treatment approaches to the first 6 months of treatment will be published as frameworks for use. Coupled with standardized data collection at routine intervals, use of these treatment plans will serve as the basis for rigorous study of comparative effectiveness of the regimens as used in clinical practice.

## Disclosure

Esi M. Morgan DeWitt: None; Timothy Beukelman: None; Peter A. Nigrovic: None; Karen Onel: None; Sampath Prahalad: None; Rayfel Schneider: Hoffmann-La Roche, Inc., 5, 9; Matthew Stoll: None; Carol A. Wallace: Amgen Inc., 2, Centocor, Inc., 2, Pfizer Inc, 2; Yukiko Kimura: Genentech, Inc., 5.

